# Medicines for Anxiety: A Hundred Years of Tranquillity and More to Come?

**DOI:** 10.1002/hup.70034

**Published:** 2026-02-20

**Authors:** David Nutt

**Affiliations:** ^1^ Division of Psychiatry, Neuropsychopharmacology Imperial College London London UK

The modern use of medicines to treat anxiety began in the 1930s when barbiturates began to take over from bromide salts for tranquilisation on the grounds of improved tolerability [Glatt 1962]. Their use expanded after World War Two, often in combination with amphetamines (e.g., ‘Purple Hearts’), until in the 1960s concerns about their toxicity in accidental overdose (e.g., the case of the actress Marilyn Monroe) plus dependence, led to the search for safer alternatives. As a medical student in Guy's hospital in the early 1970s my interest in psychiatry was boosted by caring for several barbiturate‐dependent patients undergoing withdrawal, and being intrigued by their florid hallucinations and delusions. Barbiturate replacements included methaqualone, glutethimide and clomethiazole, but these too had issues of overdose toxicity, dependence and withdrawal [Cowen and Nutt 1982]. In the early 1970s barbiturates were sufficiently commonly prescribed for a national campaign to stop them being instigated with the rather clever acronym CURB (Campaign to Use Restrict Barbiturates). This succeeded largely because much safer alternatives were becoming available in the 1960s ‐ the benzodiazepines such as diazepam and chlordiazepoxide. These became the dominant anxiolytic (and hypnotic) medicines for the ensuing 3 decades because they were effective, generally well tolerated and it was virtually impossible to die from a benzodiazepine‐alone overdose.

In parallel with the discovery of the benzodiazepines in the 1950s was a less obvious but eventually more significant development for anxiety treatments—the discovery of the monoamine oxidase inhibitors (MAOIs) such as phenelzine and the tricyclic antidepressants (TCAs) such as imipramine [see footnote]. Although the MAOIs were developed for depression, UK psychiatrists of the St Thomas' school of William Sargant observed they had powerful anti‐phobic effects, for example they could treat agoraphobia [Sargant and Dally 1962]. However the (exaggerated) fear of toxicity of MAOIs, the need for dietary control (to avoid the tyramine ‘cheese’ reaction) plus their delayed onset of action made them much less easy to use than the benzodiazepines, which worked almost immediately. The use of MAOIs became limited to specialist centres whereas benzodiazepines became widely used especially in primary care.

Efficacy of TCAs for anxiety came in the early 1960s by Klein and Fink in the USA. Whilst exploring the efficacy of imipramine across several psychiatric disorders, and confirming its value in depression, they also noted a powerful effect on sudden anxiety attacks, but not on persistent worry [Klein and Fink 1962]. From this they suggested there were two types of anxiety: panic anxiety which responded to imipramine, and generalised anxiety which did not, but did respond to benzodiazepines. Despite hostile resistance by some UK ‘experts’ to this concept it has now become generally accepted.

Initially it was thought that the benzodiazepines were ineffective in panic disorder but later more potent benzodiazepines than diazepam, specifically alprazolam and clonazepam were tested and found to work. In 1981 alprazolam (‘Xanax’) was approved for panic disorder in the USA and later in the UK, though it was never available in the National Health Service. About this time the mechanism of action of benzodiazepines, barbiturates and related drugs (including bromides) was identified as potentiation of GABA ‐the brain's inhibitory (hence calming) neurotransmitter [Nutt and Malizia 2001].

By the early 1970s the pharmacological mechanisms of the MAOIs (monoamine oxidase inhibition) and TCAs (monoamine reuptake blockade) had become understood: both increase monoamine levels in the synapse. This discovery provoked a new wave of research to discover new (patentable) drugs with similar mechanisms. Within the MAOI class the significant development was moclobemide, which was the first subtype selective inhibitor: it only acts on the MAO‐A subtype of the enzyme. Such selectivity reduced the risk of dietary interactions and improved general tolerability. The utility of older MAOIs in social phobia (now renamed social anxiety disorder) had by the 1980s been well established by Versiani and colleagues in Brazil and they then showed moclobemide was similarly effective [Versiani et al. 1992] and from this it became the first medicine specifically approved for social anxiety disorder.

The evolution of imipramine into more selective monoamine reuptake blocking TCAs developed in 1970s with more serotonin‐selective ones such as clomipramine and more noradrenaline‐selective ones such as desipramine. Subsequently new selective drugs with non‐tricyclic chemical scaffolds emerged: the SSRIs (selective serotonin reuptake inhibitors) and reboxetine (a noradrenaline reuptake inhibitor). The improved safety and tolerability of these new medicines especially the SSRIs led to them changing the pattern of treatment provision for psychiatric disorders in the UK with general practitioners taking on most cases of depression rather than refer to psychiatry services.

The fact that imipramine blocked both serotonin and noradrenaline reuptake effects raised the question of which—or both?—might be responsible for its effects in panic disorder. This question was resolved when the SSRIs were found to be as effective and easier to use than imipramine, with several getting marketing authorisations. In fact, paroxetine became the first drug in the UK to be approved for panic disorder as imipramine was never submitted for approval. Subsequently various SSRIs were found efficacious in OCD, SAnD, GAD, and PTSD, with some licensed. Both BAP [Baldwin et al. 2014] and NICE anxiety guidelines now recommend them as first line pharmacological treatments for most anxiety disorders, before benzodiazepines.

Subsequently there have been several less influential developments. The first were the serotonin 5‐HT1A receptor agonists such as buspirone (UK/USA) and tandospirone (Japan) which have efficacy in generalised anxiety disorder (GAD) though not other anxiety disorders [Baldwin et al. 2014]. Gabapentin and its derivative pregabalin were developed as ‘GABA promoting’ agents for epilepsy and were subsequently found useful for GAD [Baldwin et al. 2014]. It turned out these two agents (the gabapentanoids) were wrongly named as they do not work through GABA but instead block calcium channels. Pregabalin is now widely used as a supposedly safer alternative to benzodiazepines, though evidence suggests it may in fact be more problematic, especially when used with opioids.

And what of the future? The neuroscience of GABA has evolved spectacularly since the benzodiazepines were found to have their own binding site in 1980. Now more than 16 subtypes of GABA‐A receptor sub‐units have been identified in mammalian brain each with different locations and functions. The alpha‐2 and alpha‐3 subtypes are thought to be the main mediators of the therapeutic effects of benzodiazepines [Nutt and Malizia 2001]. However all current anxiolytic benzodiazepines also activate the alpha 1 subtype which mediates both the sedative and reinforcing effects of benzodiazepines plus the development of tolerance and withdrawal. Hence current research targeting GABA for anxiety focus on drugs selective for the alpha 2 and 3 subtypes with no/minimal activity at the alpha‐1. Several have been developed with the current leading candidate being darigabat (https://clinicaltrials.gov/study/NCT04244175). Another GABA target is the neurosteroid receptor. One agonist is fasedienol, which is under development as a ‘prn’ (as required) treatment for performance anxiety (Monti et al. 2024). It is taken nasally where it activates nerves that project into the amygdala which leads to GABA release there. Finally, several companies are exploring cholinergic nicotinic alpha‐7 drugs as fast‐acting anxiolytics, based on data that show these receptors in the amygdala also have a role in anxiety control (He et al. 2025).

## 1 Footnote on Imipramine

Imipramine was developed from earlier antihistamine molecules of tricyclic structure such as promethazine that were discovered in the 1940s. Several of these antihistamines are still in use as sedatives and tranquilisers (especially for children) without modern‐level trial evidence. One, hydroxyzine has since been shown efficacious in GAD a placebo controlled study https://pubmed.ncbi.nlm.nih.gov/15949921/ though does not have marketing authorisation for this indication the UK.

## Conflicts of Interest

The author declares no conflicts of interest.

## Data Availability

Data sharing not applicable to this article as no datasets were generated or analysed during the current study.
